# Transfer of autologous mitochondria from adipose tissue-derived stem cells rescues oocyte quality and infertility in aged mice

**DOI:** 10.18632/aging.101332

**Published:** 2017-12-27

**Authors:** Zhen-Bo Wang, Jian-Xiu Hao, Tie-Gang Meng, Lei Guo, Ming-Zhe Dong, Li-Hua Fan, Ying-Chun Ouyang, Guopeng Wang, Qing-Yuan Sun, Xiang-Hong Ou, Yuan-Qing Yao

**Affiliations:** ^1^ State Key Laboratory of Stem Cell and Reproductive Biology, Institute of Zoology, Chinese Academy of Sciences, Beijing 100101, China; ^2^ University of Chinese Academy of Sciences, Beijing 100101, China; ^3^ Department of Obstetrics and Gynecology, General Hospital of Chinese People's Liberation Army, Beijing 100853, China; ^4^ Center for Reproductive Medicine, Guangdong Second Provincial General Hospital, Guangzhou 510317, China; ^5^ The Core Facilities at School of Life Sciences, Peking University, Beijing 100871, China

**Keywords:** autologous ADSCs, mitochondria, oocyte, fertility, aged

## Abstract

Elder women suffer from low or loss of fertility because of decreasing oocyte quality as maternal aging. As energy resource, mitochondria play pivotal roles in oocyte development, determining oocyte quality. With advanced maternal age, increased dysfunctions emerge in oocyte mitochondria, which decrease oocyte quality and its developmental potential. Mitochondria supplement as a possible strategy for improving egg quality has been in debate due to ethnic problems. Heterogeneity is an intractable problem even transfer of germinal vesicle, spindle, pronuclei or polar body is employed. We proposed that the autologous adipose tissue-derived stem cell (ADSC) mitochondria could improve the fertility in aged mice. We found that autologous ADSC mitochondria could promote oocyte quality, embryo development and fertility in aged mice, which may provide a promising strategy for treatment of low fertility or infertility in elder women.

## INTRODUCTION

The fertility of women decreases with maternal aging, resulting from various kinds of reasons including decreased follicle number, altered reproductive endocrinology, increased reproductive tract defect, decreased embryo quality and impaired oocyte quality [1]. Among the possibilities, decreased oocyte quality with maternal aging is the main reason because oocyte donation from young women could rescue the low live birth rate in elder women. With maternal aging, both the nuclear maturation and cytoplasmic quality are affected, and oocyte aneuploidy arising from chromosome segregation error increases dramatically [2]. The obvious change in oopalsm as maternal aging is the mitochondrial dysfunction.

It is well known that mitochondria function in energy production and apoptosis in cells. As the most prominent cell organelles in oocytes, mitochondria play pivotal functions and determine the developmental competence of oocytes [3-5]. With advanced maternal age in women, the most common aberrations in mitochondrial structure are mitochondrial swelling and cristae disruption [6). Mitochondria are the main source of ATP through oxidative phosphorylation in mammalian oocytes [7]. It is reported that reduced ATP content and metabolic level could be detected in aged oocytes, which would affect oocyte quality and embyogenesis [8]. Mitochondrial malfunction is highly related with defects in spindle organization, cell cycle progress and chromosome segregation in oocytes of aged women and mice [9,10]. Mitochondrial dysfunction is a major contributing factor for negative outcomes in IVF in general, especially in women of advanced maternal age [1]. The findings reminded the researchers that mitochondria supplement or replacement in oocytes might be a possible strategy for infertility treatment in elder women.

The mitochondria replacement by transfer of heterologous ooplasm, germinal vesicle, spindle, polar body or pronuclei has been tested in animals and humans to improve developmental potential of aged defective oocytes or to prevent trans-generational mitochondrial disease transmission, but clinical translation of these techniques requires further validation for their efficacy and safety. Especially, the compatibility between donor mtDNA and the recipients, and mitochondrial hetetroplasmy are still a concern [11]. Transfer of autologous mitochondria from cumulus and granulosa cells were tested for oocyte quality rescue [12], but it is worth noting that cumulus and granulosa cells go through aging with oocyte as maternal aging. The method of AUGMENTA was raised to use autologous oogonial stem cells (OSCs) as mitochondria resource for supplement [13], while the existence of OSCs is still a controversial issue [14-16]. We supposed that the autologous ADSCs might be an ideal mitochondrial source for rescuing oocyte quality and fertility. In our study, we found that supplement of autologous ADSC mitochondria could improve oocyte quality, embryogenesis and fertility in aged mice. We propose that autologous ADSC mitochondria supplement may be a promising strategy for fertility retrieval in women with advanced reproductive age.

## RESULTS

### Cultivation and characterization of mouse ADSCs

**Figure 1:**
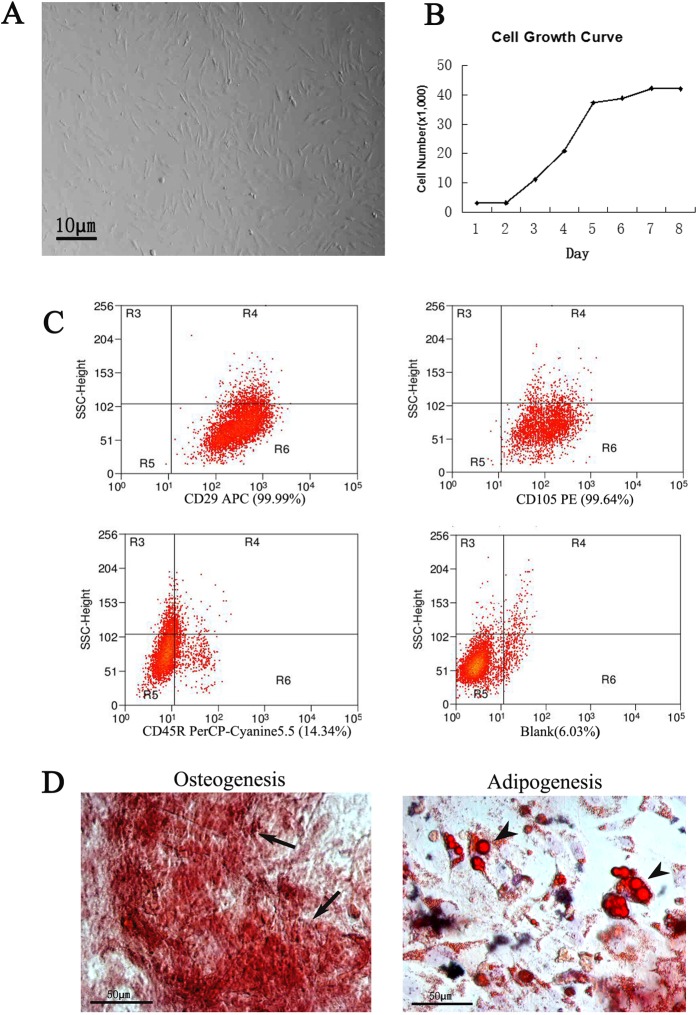
Culture and identification of ADSCs The ADSCs exhibited typical fibroblastic morphology (**A**) and normal cell growth curve (**B**), bar=10μm. (**C**) Flow cytometric analysis of ADSCs. Compared to the positive rate of blank control group (6.03%), the cells were positive expression of CD29 (99.99%), CD105 (99.64%) and negative expression of CD45R (14. 34%). (**D**) Identification of ADSCs through osteogenesis and adipogenesis. The ADSCs differentiate into osteoblasts and adipocytes. Arrows indicate osteoblasts, arrowheads indicate adipocytes.

Cells were isolated and cultured in dishes (Fig. 1A and 1B). After 4 days of culture, the cells grew to spindle-shaped and formed symmetric colonies. Fluorescent-activated cell sorting analysis was carried out to identify the expression of specific surface markers on the cells after approximately three passages. Those markers like CD29 and CD105 were expressed in more than 99% of the populations. In contrast, the majority of the adherent cells were negative for these markers (Fig. 1C). These ADSCs were multipotential, which was determined by their ability to differentiate into osteoblasts and adipocytes (Fig.1D).

### ADSC mitochondria in aged mice show normal morphology

**Figure 2:**
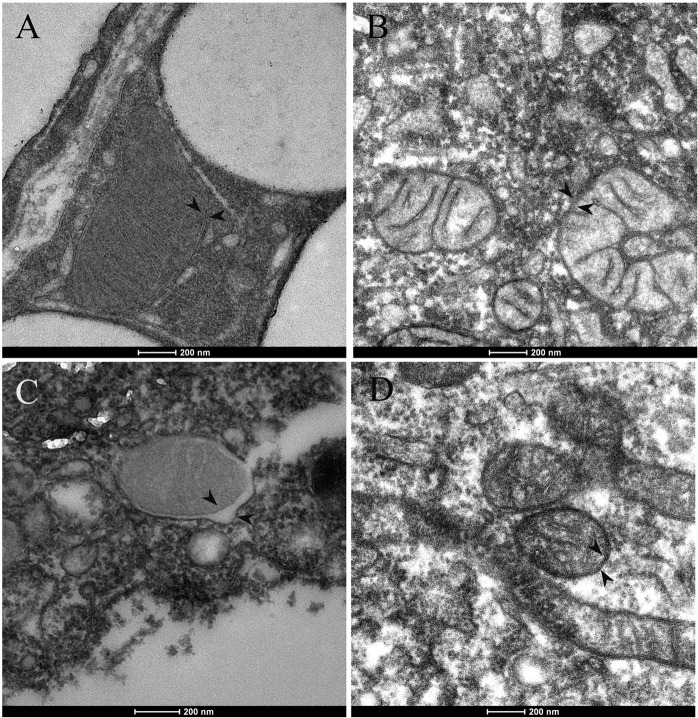
Mitochondrial ultrastructure in adipocytes and ADSCs of aged mice Mitochondria with normal cristae and morphology in adipocytes (**A**) and ADSCs (**B**) of young mice (8-week-old). (**C**) In the adipocytes of aged mice (1-year-old), alternations in mitochondrial morphology was shown. Mitochiondria had abnormal swelling and different electron densities between inner and outer mitochondrial membranes. (**D**) Mitochondrial morphology in ADSCs of old mice appeared normal. Arrowheads indicate mitochondrial inner and outer membrane.

Mitochondria play pivotal roles in oocyte development and embryogenesis. We would like to check if ADSCs could be a mitochondria resource to improve oocyte and embryo development in aged mice. First, we wanted to know whether ADSC mitochondria show normal morphology by transmission electron microscopy. Compared to mitochondria in adipose tissue in young mice, we could see the aberrant mitochondria in aged adipose tissue (Fig. 2). Interestingly, no obvious difference could be detected in ADSC mitochondrial morphology between aged and young mice.

### Autologous ADSC mitochondria restore mtDNA numbers in oocytes and improve the quality of mature oocytes in aged mouse

**Figure 3:**
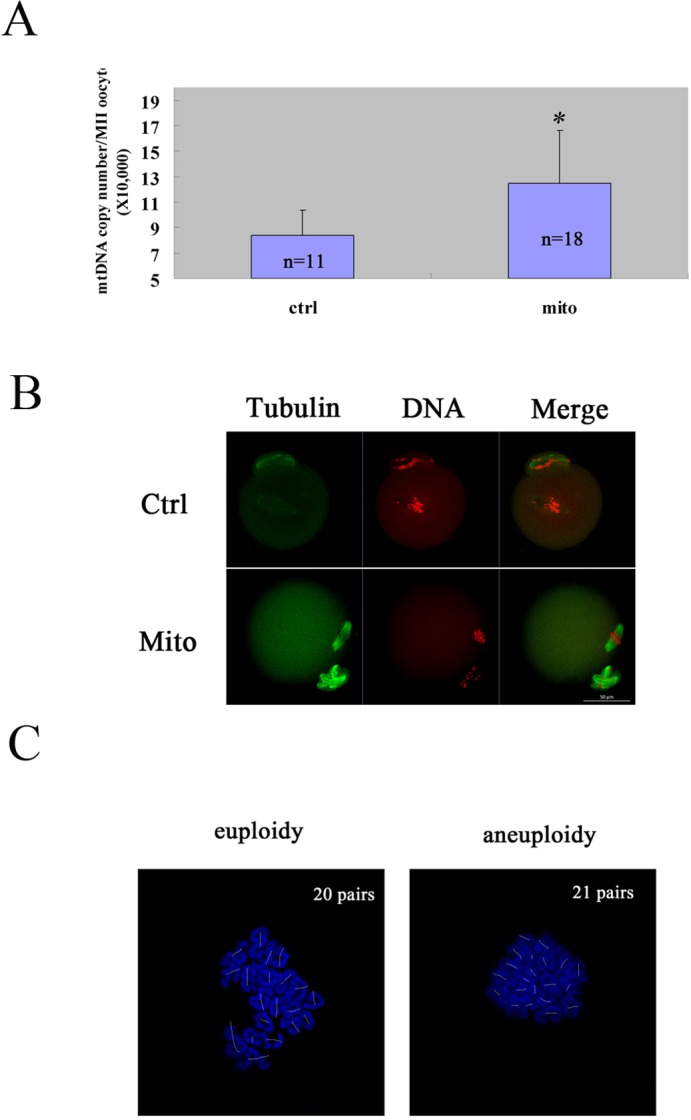
Improved oocyte quality of aged mice by autologous ADSC mitochondria supplement The GV oocytes were collected from each mouse and microinjected with mitochondria extracted from its autologous ADSCs in HTF (Mito), and the GV oocytes in control group were microinjected with HTF (Ctrl). Then the oocytes were cultured to mature in vitro, and the matured MII oocytes were checked for mtDNA numbers, spindle/chromosome orphology and karyotype. (**A**) Increased mtDNA numbers in MII oocytes of aged mice after mitochondria supplement. The mtDNA number increased dramatically after autologous ADSC mitochondria supplement compared to the control [(12.47±4.16) × 10^4^ VS (8.38±1.99) × 10^4^)]. The numbers of MII oocytes measured were indicated. (**B**) Increased normal morphology of spindle and chromosomes in MII oocytes of aged mice after mitochondria supplement. Bar=50μm. (**C**) Decreased aneuploidy in MII oocytes of aged mice after mitochondria supplement. Bar=50μm.

Then we explored the effects of autologous ADSC mitochondria transfer on oocyte quality by in vitro maturation. We microinjected the autologous ADSC mitochondria from an aged mouse into its own GV oocytes, and cultured them to mature in vitro. First, we calculated the mtDNA numbers in matured MII oocytes of the ADSC-mitochondrion -microinjected group and the control group, and we found that the mtDNA number in microinjection group was obviously increased [(12.47±4.16) × 10^4^ VS (8.38±1.99) × 10^4^] (Fig. 3A). Furthermore, the microinjection of ADSC mitochondria could promote the status of spindle organization and chromosome alignment in the equatorial plate in MII eggs from aged mice (5/5 VS 2/9) (Fig. 3B). Accordingly, the aneuploid rates of the MII eggs could be remarkably reduced (4/12 VS 11/18) (Fig. 3C).

### Autologous ADSC mitochondria promote embryo development in aged mice

**Figure 4:**
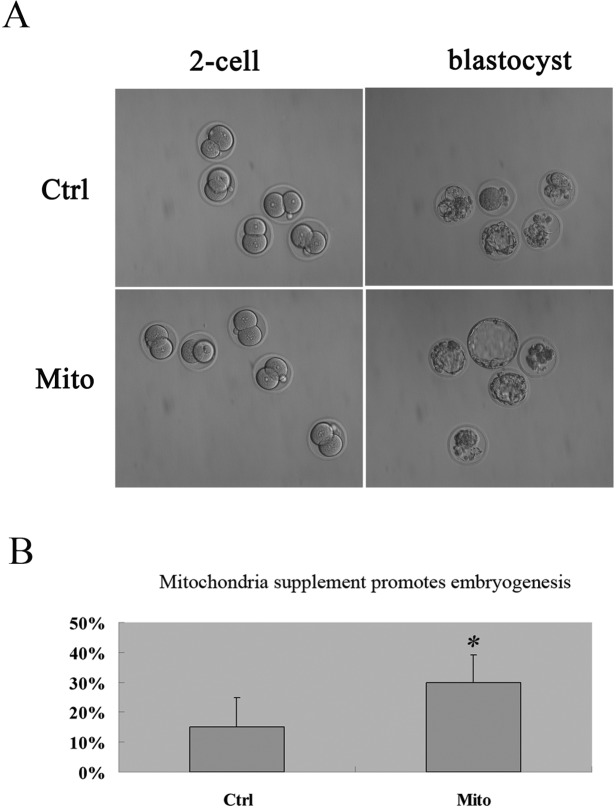
Promoted embyogenesis in aged mice through supplement of autologous ADSC mitochondria in MII oocytes MII oocytes were collected from each mouse for ICSI and microinjection with mitochondria extracted from its autologous ADSCs in HTF (Mito, n=20), and for control group, the GV oocytes were collected for ICSI and microinjected with HTF (Ctrl, n=20). Then the embryos were cultured in vitro, and the embryo development was recorded (**A**, 20X) and developmental rates was calculated (**B**), showing the improved embryogenesis after autologous ADSC mitochondria supplement in aged mice.

We carried out autologous ADSC mitochondria microinjection combining with intracytoplasmic sperm injection (ICSI) into MII oocytes to explore the effect of autologous ADSC mitochondria supplement on embryo development. Interestingly, we found that the autologous ADSC mitochondria could improve the blastocyst rates in vitro in aged mice [30% VS 15%, Fig. 4A, 4B).

### Autologous ADSC mitochondria partially rescue fertility in aged mice

Then we transferred the one-cell embryo with two pronuclei after autologous ADSC mitochondria microinjection and ICSI. Favorably, we obtained eight pups after transferring 51 embryos into 9 recipients in mitochondria transfer group, while we obtained only one pup after transferring 50 embryos into 7 recipients in control group (Table 1). So we could see the obvious affect of autologous ADSC mitochondria on fertility improvement.

**Table 1:** Autologous ADSC mitochondria supplement contributes to fertility recovery in aged mice

	Embryos (n)	Recipients (n)	Pups (n)
Mito	51	9	8
Ctrl	50	7	1

## DISCUSSION

In this study, we supposed that autologous ADSCs could be an ideal source of mitochondria to increase oocyte quality and fertility in elder women. To test this, we carried out several experiments in an aged mouse model. We found that the supplement of autologous ADSC mitochondria could reduce the aneuploidy in mature oocytes and increase embryogenesis in vitro and fertility in vivo in aged mice. Our study may provide a promising strategy to increase oocyte quality and fertility in elder women.

### Autologous tissues/cells as mitochondria source for treatment

Based on the important roles of mitochondria in oocyte and embryo development, the scientists have been trying to carry out mitochondria supplement to increase oocyte quality [21]. The researchers have realized that mitochondria supplement from xenogenic cells causes severe concerns [21]. Mitochondria from autologous tissues could avoid **t**hese problems. Autologous cumulus granulosa cells [12] and oogonial stem cells [13] have been proposed to be the mitochondria source. For cumulus granulosa cells, the problem is that the cumulus granulosa cells could be aging with oocyte as maternal aging. The existence of oogonial stem cells is still a big question. In our study, we used autologous ADSCs which are from adipose tissue as mitochondria source. The adipose tissue is rich in animals and human, and there is low if any risk to acquire.

In our experiments, we harvested the adipose tissues from anaesthetic aged mouse one by one. After In our In our experiments, we harvested the adipose tissues from anaesthetic aged mouse one by one. After acquisition of subcutaneous abdominal fat, the operative wounds were stitched. During two weeks, the mouse would heal well for oocyte collection when enough ADSC cells could be harvested for mitochondria extraction. For women, it is acceptable to acquire autologous adipose tissues by liposuction which is widely used for weight loss. So it might say that the risk for women could be as small as to ignore.

### The Warburg Effect in metabolisms of early embryo and stem cells

For glucose metabolism, it is known that the efficient way to generate ATP is that glucose is metabolized into pyruvate, which goes into tri-carboxylic acid cycle for ATP production. A metabolic adaptation called the Warburg Effect occurs in and supports rapid proliferation of the cells like early embryos [22], stem cells [23] and cancer cells [24]. For Warburg Effect, one of the characteristics is that the pyruvate is metabolized to lactate rather than into the tri-carboxylic acid cycle, indicating that glycolysis dominants in the ATP generation [25, 26]. The glycolysis could meet other critical metabolic requirements of rapid-proliferated cells, and at the same time the inefficient way for ATP generation could meet its energy requirement. It is believed that the survival of pre-implantation embryo is best met by a relatively low metabolic level [27].

The metabolic similarity in early embryo (oocyte) and stem cells are reflected in their mitochondrial morphology. Mitochondria in the oocytes are spherical with few cristae, and they began to elongate at the 4-cell to morula stages. Similarly, the inner cell mass cells contain spherical mitochondria [28]. For human embryonic stem cells, the undifferentiated cells have small mitochondria with a few cristae [29, 30]. The mitochondria show a similarly less mature form in ADSCs (Fig. 3).

## MATERIALS AND METHODS

### Animals

The six-seven-month-old ICR mice were purchased from SPF (Beijing) Biotechnology Co. Ltd., and then they were further raised for six months to be used in the experiments. Mice were maintained in alternating 12-hour light/dark cycles. Animal care and use were carried out in accordance with the Animal Research Committee guidelines of the Institute of Zoology, Chinese Academy of Sciences, China.

### Harvest of adipose tissues and ADSCs

ADSC isolation was performed according to the protocol previously reported [17]. Firstly, subcutaneous adipose tissues were harvested from the abdominal region of mice, and there was a one-to-one relationship between the tissues and the mice. Secondly, the adipose tissues were washed with sterile phosphate-buffered saline (PBS) to remove debris and red blood cells, and then the tissues were minced and digested in serum-free medium with 0.1% type I collagenase (Sigma, St. Louis, MO, USA). The digestion was performed for 30-60 minutes with gentle agitation at 37°C. Thirdly, an equal volume of DEME low-glucose medium (Gibco, USA) supplemented with 10% fetal bovine serum (FBS, Gibco, USA) was used to inactivate the enzymes, and a 200-μm mesh filter was used to filter samples. The filtered samples were centrifuged at 600 g for 5 minutes to obtain cellular pellets. Finally, the cellular pellets were cultured in DEME low-glucose medium with 10% FBS, 50 U/ml penicillin, and 50μg/ml streptomycin in a humidified incubator at 37°C with 5% CO_2_.

### ADSC identification and flow cytometry

Flow cytometry was used to analyze the surface-marker expression of ADSCs by the following antibodies: CD29, CD105 and CD45R (e Bioscience, USA). 0.25% trypsin-EDTA, neutralized with FBS-containing culture medium was used to treat and detach the adherent cells, and single cells were obtained by pipetting. The cells were incubated with the above antibodies for 30 minutes at 4°C, washed twice with PBS, resuspended in 0.5 ml PBS, and immediately analyzed by using a MoFlo XDP flow cytometer (Beckman, USA). A minimum of 2 × 10^5^ cells were used for each sample.

### Osteogenic and adipogenic differentiation

For osteogenic differentiation, cells were seeded at a density of 2 × 10^3^ cells/cm^2^ onto the 24-well plate. After 24 hours culture, the medium was replaced by osteogenic differentiation medium, and the cells were cultured for 3 weeks. Then the cells could be fixed and stained with Alizarin red. For osteogenic differentiation, cells were seeded at a density of 2 × 10^3^ /cm^2^. After confluence, cells were incubated in adipogenic differentiation medium for 2 weeks. Then the cells were fixed and stained with Oil Red O. For both the experiments, the differentiation medium was changed every 3 days.

### Mitochondria extraction and microinjection

Mitochondria were prepared by differential centrifugation. The ADSCs were prepared with HTF at 10^7^ cells per ml. Then the cells were treated 4-6 times with a cell homogenizer on ice. The homogenate was centrifuged for 20 min at 2000r/m, the supernatant was collected and centrifuged for 20 min at 10000r/m again. The sediments were resuspended very slowly with 1μL HTF. That is, the mitochondria extracted from 10^7^ ADSCs were in 1uL HTF solution. All the processes were carried out at 4°C, and the extracts were maintained at 4°C for microinjection. For the GV oocytes, 7-8pL mitochondrial in HTF were micro-injected into each oocyte using a Nikon Diaphot ECLIPSE TE 300 (Nikon UK Ltd., UK) inverted microscope equipped with Narishige MM0202N hydraulic three-dimensional micromanipulator (Narishige Inc., USA) and microinjection was completed within 30 minutes. For MII oocytes, 10pL mitochondria were microinjected into each oocyte combing ICSI (for details, please see “ICSI and embryo transfer” below).

### Transmission electron microscopy

The adipose tissues were dissected and fixed with 2.5% (vol/vol) glutaraldehyde in 0.2 M cacodylate buffer overnight. After washing in 0.2 M PBS, the tissues were cut into small pieces of approximately 1 mm^3^ and immersed in 1% OsO_4_ in 0.2 M cacodylate buffer for 2 h at 4°C. Then, the samples were dehydrated through a graded ethanol series and embedded in resin (Low Viscosity Embedding Media Spurr's Kit, EMS, 14300). ADSCs were fixed in 2.5% glutaraldehyde in 0.1 M PBS (pH 7.4) overnight at 4°C. After being washed with PBS, the samples were post-fixed with 1% osmium tetroxide containing 0.8% potassium ferricyanide at room temperature for 1 h, embedded in Spurr's resin. Ultrathin sections were cut on an ultramicrotome, stained with uranyl acetate and lead citrate, and observed using FEI Tecnai F20 transmission electron microscope (Oregon, USA).

### Oocyte collection and culture

For in vitro maturation, the GV oocytes were collected and cultured in M16 medium under liquid paraffin oil at 37°C in an atmosphere of 5% CO_2_ in air. To obtain MII oocytes, mice were induced to superovulate by injection of 8 IU PMSG followed 48 h later by injection of 8 IU hCG. 14 to 15 h after hCG injection, mice were sacrificed and the oviductal ampullae were broken to release the cumulus–oocyte complexes. MII oocytes were freed of cumulus cells by exposure to 300μg/ml hyaluronidase (Sigma, St. Louis, MO, USA). The oocytes were collected for microinjection, immunofluorescent staining and ICSI.

### ICSI and embryo transfer

ICSI was carried out as described previously [18]. After treatment, sperm heads were transferred into a mitochondria-HTF drop and used for ICSI. Briefly, a single sperm head was sucked into an injection pipette that was attached to a piezo-electric driving unit (Model PMAS-CT150; Prime Tech Ltd., Japan). A mouse MII oocyte was stabilized using a holding pipette and the zona pellucida was penetrated by applying several piezo pulses. After injection, oocytes were washed three times in HTF and incubated at 37°C under 5% CO2.

### Measurement of mitochondrial DNA copy numbers by quantitative real-time PCR

Quantitative real-time PCR procedures for mitochondrial DNA (mtDNA) numbers have been described previously [19). The mouse mtDNA-specific primers: B6 forward, AACCTGGCACTGAGTCACCA, and B6 reverse, GGTCTGAGTGTATATATCATGAAGAGAAT were used to make the external standard for absolute quantification of mtDNA [20]. The PCR products were ligated into the T-vector. A standard curve was produced by seven 10-fold serial dilutions of purified plasmid standard DNA. Briefly, one oocyte was loaded at the bottom of a PCR tube with 5 ml lysis buffer and incubated at 55°C for 2 h. After heat inactivated at 95°C for 10 min, the samples were used for PCR analysis. Quantitative real-time PCR was performed using the ABI system and mouse mtDNA-specific primers. Linear regression analysis of all standard curves for samples showed a correlation coefficient higher than 0.97. All measurements were performed in triplicates.

### Immunofluorescence and confocal microscopy

For single staining of α-tubulin, oocytes were fixed in 4% paraformaldehyde in phosphate-buffered saline (PBS, pH 7.4) for at least 30 min at room temperature. After being permeabilized with 0.5% Triton X-100 at room temperature for 20 min, oocytes were blocked in 1% bovine serum albumin-supplemented PBS for 1 h and incubated overnight at 4°C with 1:200 anti-α-tubulin-fluorescein isothiocyanate antibody. After three washes in PBS containing 0.1% Tween-20 and 0.01% Triton X-100, the oocytes were stained with propidium iodide (PI) (10μg/ml in PBS). Then, the oocytes were mounted on glass slides and examined with a confocal laser scanning microscope (Zeiss LSM 780, Berlin, Germany).

### Statistics

The percentages from at least three repeated experiments were expressed as mean ± SEM. Data were analyzed by paired-samples t-test. *P* < 0.05 was considered statistically significant.
